# RETRACTION: lncRNA NEAT1/miR‐495‐3p Regulates Angiogenesis in Burn Sepsis Through the TGF‐β1 and SMAD Signaling Pathways

**DOI:** 10.1002/iid3.70177

**Published:** 2025-03-21

## Abstract

**RETRACTION**: Y. Meng, Z. Hao, H. Zhang, P. Bai, W. Guo, X. Tian and J. Xu, “lncRNA NEAT1/miR‐495‐3p Regulates Angiogenesis in Burn Sepsis Through the TGF‐β1 and SMAD Signaling Pathways,” *Immunity, Inflammation and Disease* 11, no. 1 (2023): e758, https://doi.org/10.1002/iid3.758.

The above article, published online on January 16, 2023 in Wiley Online Library (wileyonlinelibrary.com), has been retracted by agreement the journal Editor‐in‐Chief, Marc Veldhoen; and John Wiley & Sons Ltd. The retraction has been agreed due scientific flaws and inconsistencies found in the methodology and results of this article. While the authors were able to provide some supporting data, this was not sufficient and the irregularities remain. The editors have lost confidence in the results and conclusions presented in this study. The authors disagree with the retraction.

**RETRACTION**: Y. Meng, Z. Hao, H. Zhang, P. Bai, W. Guo, X. Tian and J. Xu, “lncRNA NEAT1/miR‐495‐3p Regulates Angiogenesis in Burn Sepsis Through the TGF‐β1 and SMAD Signaling Pathways,” *Immunity, Inflammation and Disease* 11, no. 1 (2023): e758, https://doi.org/10.1002/iid3.758.

The above article, published online on January 16, 2023 in Wiley Online Library (wileyonlinelibrary.com), has been retracted by agreement the journal Editor‐in‐Chief, Marc Veldhoen; and John Wiley & Sons Ltd. The retraction has been agreed due scientific flaws and inconsistencies found in the methodology and results of this article. While the authors were able to provide some supporting data, this was not sufficient and the irregularities remain. The editors have lost confidence in the results and conclusions presented in this study. The authors disagree with the retraction.

